# Supplementary material for: Trait variation in patchy landscapes: Morphology of spotted salamanders (Ambystoma maculatum) varies more within ponds than between ponds
Source: PLoS One. 2024 Apr 4;19(4):e0299101. doi: 10.1371/journal.pone.0299101 (PMC10994278; doi:10.1371/journal.pone.0299101)
Supplement: S1 File — Supporting information for the manuscript contains additional information on pond characteristics, tables reporting outputs of statistical models, and data on the invertebrates. (DOCX) [file pone.0299101.s001.docx]

Supplementary Material for:

**Trait variation in patchy landscapes: morphology of spotted salamanders (*Ambystoma maculatum*) varies more within ponds than between ponds**

Elizabeth T. Green^1,2^, Anthony I. Dell^1,3^, John A. Crawford^1^, Elizabeth G. Biro^3,4^, David R. Daversa^1,3,5*^

^1^ National Great Rivers Research and Education Center (NGRREC), East Alton, IL 62024, USA

^2^ Department of Biology, University of North Carolina at Chapel Hill, Chapel Hill, NC 27599, USA

^3^ Department of Biology, Washington University in St. Louis, St. Louis, MO 63130, USA

^4^ Tyson Research Center, Washington University in St. Louis, St. Louis, MO 63130, USA

^5^ La Kretz Center for California Conservation Science, Institute of the Environment and Sustainability, University of California, Los Angeles, Los Angeles, CA, 90095, USA

**Pond Characteristics**

Although not a focus of the study, we measured several environmental variables of the six focal ponds. To estimate relative densities of spotted salamander larvae, we systematically dip-netted ponds (1 dip-net/3 m^2^) before collection. We installed iButtons into each pond 0.5-1 meter from the shoreline, within the range of the pond where we dipnetted for salamanders. The iButtons recorded temperature once/hour over the course of the survey period. We also surveyed ponds for invertebrate and vertebrate predators of *A. maculatum* using a 30 cm-wide, 1 mm mesh dip-net, twenty net sweeps approximately 1 meter long were conducted throughout the pond at varying depths and locations in the summer of 2013 (2 ponds) and 2015 (4 ponds). Ponds sampled in both 2013 and 2015 were compared to confirm the consistency of sampling. Samples were identified in the field if possible. All unidentifiable individuals were preserved in 70% ethanol for subsequent identification. We used a dissecting microscope for species identification to the lowest possible taxonomic group according to the keys of Needham, Westfall Jr, & May (2000); Merritt, Cummins, & Berg (Merritt, Cummins and Berg, 2008); Thorp & Covich (Thorp and Covich, 2009). Predator species were defined as such if there was previous literature documenting a predator-prey interaction with larval *A. maculatum*. We kept HOBO temperature loggers in ponds for the duration of the study period to estimate mean water temperature. Summary statistics for the environmental variables are reported in S1 File Table 1.

| **pond** | **age** | **canopy cover (%)** | **conspecific density (per m^2^)** | **predator density (per m2)** | **mean water temp. (Celcius)** |
| --- | --- | --- | --- | --- | --- |
| Salamander | old | 91 | 0.7 | 0.3 | 22.8 |
| Shaw | old | NA | 1.2 | 1.9 | 21.8 |
| Mincke | new | 92 | 6.5 | 2.0 | 22.4 |
| Arthur Christ | new | 94 | 1.0 | 11.5 | 22.3 |
| Forest 44 | old | 57.23 | 3.6 | 3.1 | 22.3 |
| Beth's | new | 56.97 | 1.5 | 10.0 | 22.2 |

**Table S1-1. Environmental characteristics of the six focal ponds of the study.** Pond age reflects differences in histories; ‘new’ ponds were constructed as part of a separate study in 2008, whereas ‘old’ ponds have been present for much longer and are more naturalized than ‘new’ ponds.

| **trait** | **pond** | **mean** | **sd** | **min** | **max** | **CV** |
| --- | --- | --- | --- | --- | --- | --- |
| mass | Salamander | 0.65 | 0.19 | 0.25 | 0.93 | 28.82 |
|  | Shaw | 0.46 | 0.13 | 0.16 | 0.78 | 28.39 |
|  | Mincke | 0.44 | 0.17 | 0.17 | 0.82 | 38.18 |
|  | Arthur Christ | 0.30 | 0.13 | 0.09 | 0.62 | 41.71 |
|  | Forest 44 | 0.43 | 0.14 | 0.18 | 0.80 | 31.44 |
|  | Beth's | 0.60 | 0.16 | 0.35 | 0.85 | 26.97 |
| body condition | Salamander | 0.49 | 0.10 | 0.25 | 0.92 | 21.25 |
|  | Shaw | 0.45 | 0.05 | 0.29 | 0.57 | 12.11 |
|  | Mincke | 0.45 | 0.11 | 0.13 | 0.77 | 23.28 |
|  | Arthur Christ | 0.42 | 0.06 | 0.33 | 0.64 | 14.77 |
|  | Forest 44 | 0.40 | 0.04 | 0.31 | 0.58 | 10.47 |
|  | Beth's | 0.37 | 0.06 | 0.32 | 0.54 | 15.17 |
| head length | Salamander | 7.71 | 1.24 | 4.29 | 9.83 | 16.05 |
|  | Shaw | 7.95 | 0.94 | 6.02 | 9.87 | 11.84 |
|  | Mincke | 7.74 | 1.19 | 4.67 | 9.99 | 15.36 |
|  | Arthur Christ | 6.71 | 1.19 | 3.69 | 9.15 | 17.69 |
|  | Forest 44 | 7.33 | 1.07 | 4.47 | 9.72 | 14.62 |
|  | Beth's | 8.52 | 1.05 | 6.95 | 10.69 | 12.36 |
| body length | Salamander | 14.42 | 2.18 | 9.84 | 18.22 | 15.09 |
|  | Shaw | 12.86 | 1.78 | 7.29 | 17.72 | 13.81 |
|  | Mincke | 12.90 | 2.22 | 7.68 | 18.43 | 17.22 |
|  | Arthur Christ | 11.56 | 2.13 | 6.48 | 17.26 | 18.44 |
|  | Forest 44 | 13.98 | 2.11 | 9.51 | 19.46 | 15.09 |
|  | Beth's | 16.36 | 2.07 | 13.50 | 19.42 | 12.64 |
| tail length | Salamander | 22.42 | 4.15 | 11.33 | 29.18 | 18.54 |
|  | Shaw | 19.26 | 2.92 | 10.40 | 25.60 | 15.16 |
|  | Mincke | 18.41 | 3.45 | 11.14 | 25.90 | 18.72 |
|  | Arthur Christ | 15.93 | 3.63 | 8.58 | 25.32 | 22.81 |
|  | Forest 44 | 19.43 | 2.87 | 11.06 | 27.27 | 14.77 |
|  | Beth's | 23.49 | 3.07 | 18.00 | 28.13 | 13.05 |
| total length | Salamander | 44.55 | 6.45 | 26.32 | 53.94 | 14.48 |
|  | Shaw | 40.05 | 4.64 | 24.90 | 49.33 | 11.58 |
|  | Mincke | 39.05 | 6.33 | 26.53 | 51.57 | 16.20 |
|  | Arthur Christ | 34.20 | 6.35 | 21.58 | 47.84 | 18.55 |
|  | Forest 44 | 40.74 | 5.10 | 26.91 | 53.21 | 12.53 |
|  | Beth's | 48.37 | 5.38 | 39.31 | 57.92 | 11.13 |

**Table S1-2.** **Pond-level summary statistics for salamander mass, body condition, and length (head, body, tail, total).** Summary statistics of 2016 survey data of late-phase larval salamander populations in Missouri. Sd – standard deviation, min = minimum value, max = maximum value, CV = coefficient of variation. Body condition was calculated using the scaled mass index.

| **Trait** | **Pond name** | **mass regression equation** |
| --- | --- | --- |
| **head length** | Forest 44 | y = 0.96x -1.21 |
|  | Shaw | y = 1.21x -1.44 |
|  | Salamander | y = 0.93x -1.03 |
|  | Arthur Christ | y = 1.54x -1.82 |
|  | Beth's | y = 0.79x -0.96 |
|  | Mincke | y = 1.57x -1.78 |
|  | **Overall** | **y = 1.52x -1.71** |
| **body length** | Forest 44 | y = 1.83x -2.47 |
|  | Shaw | y = 1.66x -2.18 |
|  | Salamander | y = 1.70x -2.17 |
|  | Arthur Christ | y = 2.00x -2.67 |
|  | Beth's | y = 1.65x -2.24 |
|  | Mincke | y = 1.71x -2.28 |
|  | **Overall** | **y = 1.94x -2.54** |
| **tail length** | Forest 44 | y = 1.81x -2.71 |
|  | Shaw | y = 1.54x -2.32 |
|  | Salamander | y = 1.17x -1.78 |
|  | Arthur Christ | y = 1.72x -2.60 |
|  | Beth's | y = 1.92x -2.86 |
|  | Mincke | y = 1.64x -2.46 |
|  | **Overall** | **y = 1.74x -2.6** |
| **total length** | Forest 44 | y = 2.36x -4.18 |
|  | Shaw | y = 2.3x -4.04 |
|  | Salamander | y = 1.81x -3.17 |
|  | Arthur Christ | y = 2.17x -3.87 |
|  | Beth's | y = 2.18x -3.91 |
|  | Mincke | y = 1.95x -3.49 |
|  | **Overall** | **y = 2.23x -3.94** |

**Table S1-3.** **Length-mass regression equations for salamander morphology.** Equations for regression lines expressing the relationship between mass with the length of salamander heads, bodies, tails, and the three body segments combined (total length). Both length and mass values were log-transformed in linear models used to calculate intercept and slope values for the regression lines.

| **Invertebrate Species** | **Predatory (Y/N)** | **Beth's**  **pond** | **Mincke**  **pond** | **Salamander**  **pond** | **Shaw**  **pond** | **Arthur**  **Christ**  **pond** | **Forest**  **44**  **pond** | **total** |
| --- | --- | --- | --- | --- | --- | --- | --- | --- |
| *Acilius fraternus* | yes | 0 | 0 | 1 | 0 | 0 | 0 | 1 |
| Acilius sp. larvae | yes | 0 | 3 | 0 | 0 | 0 | 0 | 3 |
| *Aeshna umbrosa* | yes | 0 | 0 | 1 | 0 | 0 | 0 | 1 |
| Agabus sp. larvae | yes | 2 | 1 | 0 | 0 | 0 | 0 | 3 |
| *Anaxyrus americanus* | no | 114 | 0 | 0 | 0 | 0 | 0 | 114 |
| Chaoborus sp. larvae | no | 108 | 38 | 0 | 0 | 0 | 0 | 146 |
| Chauliodes sp. larvae | yes | 0 | 0 | 5 | 0 | 0 | 0 | 5 |
| Chironomid sp. | no | 89 | 153 | 0 | 0 | 0 | 0 | 242 |
| Enallagma sp. | no | 1 | 0 | 0 | 0 | 1 | 0 | 2 |
| *Erythemis simplicollis* | yes | 0 | 0 | 0 | 0 | 5 | 0 | 5 |
| *Gyraulus parvus* | no | 0 | 3 | 0 | 0 | 0 | 0 | 3 |
| *Helisoma trivolvis* | no | 0 | 2 | 0 | 0 | 0 | 0 | 2 |
| Helobdella sp. | no | 0 | 7 | 0 | 0 | 0 | 0 | 7 |
| Hesperocorixa sp. | no | 0 | 0 | 1 | 0 | 0 | 0 | 1 |
| Hydrobiomorpha sp. | no | 0 | 0 | 1 | 0 | 0 | 0 | 1 |
| *Hyla versicolor* | no | 0 | 4 | 0 | 0 | 0 | 0 | 4 |
| *Laccophilus maculosa* | no | 1 | 2 | 0 | 0 | 0 | 1 | 4 |
| Laccophilus sp. larvae | no | 0 | 2 | 0 | 0 | 0 | 0 | 2 |
| *Libellula luctuosa* | yes | 7 | 0 | 0 | 0 | 0 | 0 | 7 |
| *Libellula pulchella* | yes | 4 | 0 | 0 | 0 | 0 | 0 | 4 |
| *Musculium transversum* | no | 0 | 200 | 0 | 0 | 0 | 0 | 200 |
| *Notonecta irrorata* | yes | 0 | 0 | 0 | 3 | 3 | 14 | 20 |
| *Notophthalamus viridescens louisianensis* | yes | 1 | 0 | 0 | 0 | 0 | 0 | 1 |
| Ogliochaete | no | 0 | 2 | 0 | 0 | 0 | 0 | 2 |
| *Pachydiplax longipennis* | yes | 5 | 0 | 0 | 2 | 15 | 0 | 22 |
| *Physa heterostropha* | no | 57 | 13 | 0 | 0 | 0 | 0 | 70 |
| *Pseudacris triseriata* | no | 0 | 4 | 0 | 0 | 0 | 0 | 4 |
| Pseudosuccinea sp. | no | 1 | 0 | 0 | 0 | 0 | 0 | 1 |
| *Rana clamitans* | yes | 3 | 0 | 0 | 25 | 0 | 0 | 28 |
| *Tropisternus blachelyi* | yes | 0 | 0 | 0 | 0 | 0 | 16 | 16 |
| Tropisternus sp. 1 | yes | 0 | 0 | 0 | 0 | 0 | 1 | 1 |
| Tropisternus sp. 4 | yes | 0 | 0 | 1 | 1 | 0 | 0 | 2 |
| Tropisternus sp. larvae | no | 0 | 0 | 0 | 0 | 0 | 3 | 3 |

**Table S1-4.** Counts of different species of invertebrates of focal ponds where salamanders were collected. Counts were performed within the same time period – July-August 2016 - as when salamander collected was executed, except for Beth’s pond. Counts for Beth’s pond come from a 2013 survey.

**Fig S1-1. Mean temperatures of focal ponds for the study period.** Temperatures were recorded using ibuttons placed 0.5-1 meter from the shoreline, in the area of ponds that were dipnetted. Bold lines denote the mean, box limits denote the standard error of the mean, and error bars denote the 95% confidence intervals.
