# Supplementary material for: Trait variation in patchy landscapes: Morphology of spotted salamanders (Ambystoma maculatum) varies more within ponds than between ponds
Source: PLoS One. 2024 Apr 4;19(4):e0299101. doi: 10.1371/journal.pone.0299101 (PMC10994278; doi:10.1371/journal.pone.0299101)
Supplement: S2 File — A visualization of the landmarks used for the morphometric characterizations as well as a table reporting the variance explained by principle components is shown. (DOCX) [file pone.0299101.s002.docx]

Supplementary Material for:

**Trait variation in patchy landscapes: morphology of spotted salamanders (*Ambystoma maculatum*) varies more within ponds than between ponds**

Elizabeth T. Green^1,2^, Anthony I. Dell^1,3^, John A. Crawford^1^, Elizabeth G. Biro^3,4^, David R. Daversa^1,3,5*^

^1^ National Great Rivers Research and Education Center (NGRREC), East Alton, IL 62024, USA

^2^ Department of Biology, University of North Carolina at Chapel Hill, Chapel Hill, NC 27599, USA

^3^ Department of Biology, Washington University in St. Louis, St. Louis, MO 63130, USA

^4^ Tyson Research Center, Washington University in St. Louis, St. Louis, MO 63130, USA

^5^ La Kretz Center for California Conservation Science, Institute of the Environment and Sustainability, University of California, Los Angeles, Los Angeles, CA, 90095, USA

**S2: Further information on salamander morphometrics**

**
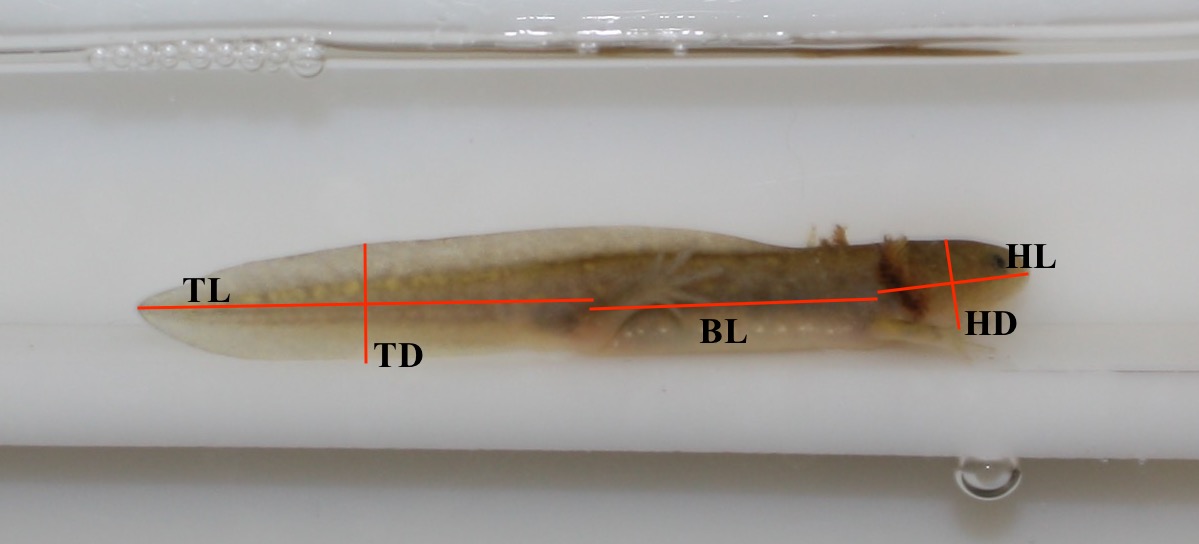
**

**(b)**


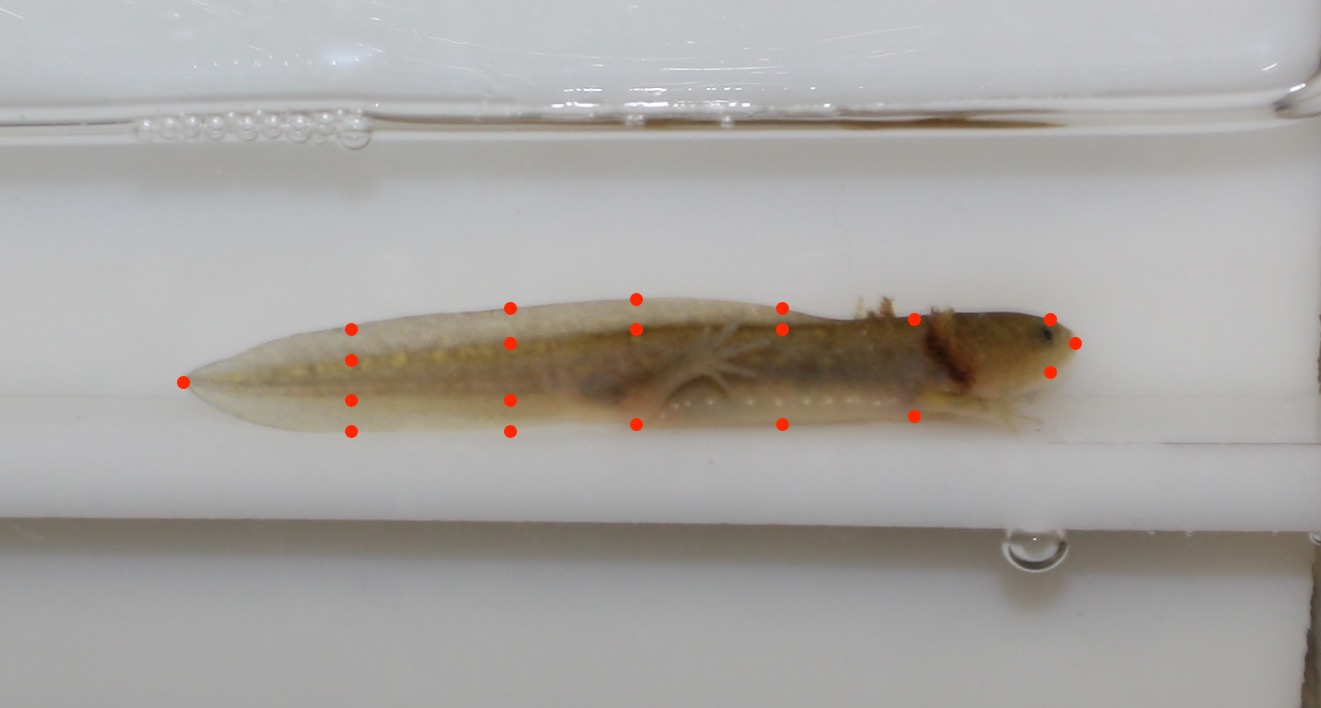


**Fig S3-1.** Morphometric analysis of larval *A. maculatum* included both **(a)** measurements and **(b)** landmarks placed for shape analysis. Linear measurements included head length (HL), maximum head depth (HD), body length (BL), tail length (TL), and mid tail depth (TD). Geometric analysis involved placing twenty landmarks to outline the shape of, and included: tip of the snout (1) above the eye (2), below the eye (3), above vent (4), below vent (5), 50% length of body (6, 7, 8), in line with hind leg (9, 10, 11), at 25% length of tail (12, 13, 14, 15), at 50% length of tail (16, 17, 18, 19), and tip of tail (20).

| **Shape** | **PC1 variance explained** | **PC2 variance explained** | **PC3 variance explained** | **PC4 variance explained** | **PC5 variance explained** |
| --- | --- | --- | --- | --- | --- |
| head | 0.372 | 0.271 | 0.149 | 0.074 | 0.070 |
| body | 0.332 | 0.249 | 0.105 | 0.102 | 0.052 |
| tail | 0.338 | 0.177 | 0.101 | 0.083 | 0.074 |
| combined | 0.271 | 0.263 | 0.101 | 0.06 | 0.048 |

**Table S3-1.** Proportion of variance explained by the first five PCs for salamander shapes.
