# Supplementary material for: Trait variation in patchy landscapes: Morphology of spotted salamanders (Ambystoma maculatum) varies more within ponds than between ponds
Source: PLoS One. 2024 Apr 4;19(4):e0299101. doi: 10.1371/journal.pone.0299101 (PMC10994278; doi:10.1371/journal.pone.0299101)
Supplement: S3 File — Detailed methods and results on the analysis of the extent to which pond age influence salamander morphology. The file includes one table reporting model outputs and two figures visualizing the observed patterns. (DOCX) [file pone.0299101.s003.docx]

Supplementary Material for:

**Trait variation in patchy landscapes: morphology of spotted salamanders (*Ambystoma maculatum*) varies more within ponds than between ponds**

Elizabeth T. Green^1,2^, Anthony I. Dell^1,3^, John A. Crawford^1^, Elizabeth G. Biro^3,4^, David R. Daversa^1,3,5*^

^1^ National Great Rivers Research and Education Center (NGRREC), East Alton, IL 62024, USA

^2^ Department of Biology, University of North Carolina at Chapel Hill, Chapel Hill, NC 27599, USA

^3^ Department of Biology, Washington University in St. Louis, St. Louis, MO 63130, USA

^4^ Tyson Research Center, Washington University in St. Louis, St. Louis, MO 63130, USA

^5^ La Kretz Center for California Conservation Science, Institute of the Environment and Sustainability, University of California, Los Angeles, Los Angeles, CA, 90095, USA

**Preliminary assessment of the influence of pond age on salamander morphology**

*Methods*

There was a distinct dichotomy among our focal ponds in their age and history. Three ponds (Mincke Pond, Arthur Christ Pond, Beth’s Pond) were constructed in 2008 for research purposes, whereas the other three ponds (Salamander Pond, Forest 44 Pond, Shaw Pond) in our sample were older and more naturalized. To capitalize on this dichotomy, we performed a preliminary assessment of whether salamander mass, length, mass-length co-variation, and shape were influenced by the age of ponds. We consider these assessments preliminary because of the low replication of ponds in our sample (N = 6). We used historical information described above to classify pond age as ‘new’ (N = 3; Mincke Pond, Arthur Christ Pond, Beth’s Pond) or ‘old’ (N = 3; Salamander Pond, Forest 44 Pond, Shaw Pond) (S1 File: Table S1-1).

*Results*

Pond age did not influence salamander mass or any measures of length (Table S2-1). However, pond age did influence certain mass-length relationships and body shapes (Figs S2-1 & S2-2, Table S2-1). Pond age influenced the scaling of mass with head and tail length (Figs S2-1 & S2-2, Table S2-1) as well as the overall shape of salamanders (Table S2-1).

| **body segment** | **df** | **AIC** | **Χ^2^** | **p** |
| --- | --- | --- | --- | --- |
| **mass & body condition** | | | | |
| mass | 1 | -387.28 | 0.58 | 0.446 |
| condition | 1 | -987.42 | 0.78 | 0.378 |
| **length** | | | | |
| head | 1 | 1336.30 | 0.02 | 0.885 |
| body | 1 | 1876.00 | 0.04 | 0.838 |
| tail | 1 | 2286.20 | 0.38 | 0.534 |
| combined | 1 | 2746.80 | 0.17 | 0.676 |
| **mass:length co-variation** | | | | |
| head | **1** | **-510.20** | **9.10** | **0.003** |
| body | 1 | -835.37 | 1.07 | 0.300 |
| tail | **1** | **-884.12** | **3.90** | **0.048** |
| combined | 1 | -1058.70 | 0.85 | 0.357 |
| **shape - PC1** | | | | |
| head | 1 | -1273.40 | 0.13 | 0.715 |
| body | 1 | -1379.00 | 2.66 | 0.103 |
| tail | 1 | -1353.90 | 0.95 | 0.330 |
| combined | **1** | **-1710.10** | **9.80** | **0.002** |
| **shape - PC2** | | | | |
| head | 1 | -1396.70 | 0.01 | 0.917 |
| body | 1 | -1517.00 | 0.09 | 0.764 |
| tail | 1 | -1618.80 | 3.19 | 0.074 |
| combined | 1 | -1728.40 | 0.20 | 0.655 |

**Table S2-1. Influence of pond age and predator density on salamander morphology.** Outputs of likelihood ratio tests of the influence of pond age on salamander morphological traits are reported. Tests were performed using GLMMs with pond age as a fixed effect and pond of capture as a random intercept term. Df = degrees of freedom, AIC = Akaike’s Information Criterion. Cases where inclusion of pond age significantly improved model fit to the data are highlighted in bold.

**Fig S2-1. The effect of pond age on mass-length relationships.** The slope values for co-variation of salamander mass with (a) head length, (b) body length, (c) tail length, and (d) total length are displayed according to the age ponds; black bars denote ‘new’ ponds that were constructed in 2008, and grey bars denote ‘old’ ponds were constructed in the mid-1900s (specific construction histories vary) and exhibited more naturalized conditions. Error bars denote 95% confidence intervals.

**Fig S2-2. The effect of pond age on mass-length relationships.** Scaling of salamander mass with (a) head length, (b) body length, (c) tail length, and (d) total length are displayed, with regression lines drawn separately for new (blue) and old (yellow) ponds. New ponds were constructed in 2008. Old ponds were constructed in the mid-1900s. Shaded areas denote 95% confidence intervals.
